# Supplementary material for: Multiple Imputation for Longitudinal Data: A Tutorial
Source: Stat Med. 2025 Jan 23;44(3-4):e10274. doi: 10.1002/sim.10274 (PMC11755704; doi:10.1002/sim.10274)
Supplement: Supplementary file 1 — Data S1 Supporting Information. [file SIM-44-0-s001.docx]

**Supplementary files**

# S1: CATS case study analysis results based on available cases.

Table S1: Point estimate (and standard error) for the effect of early depressive symptoms on subsequent standardized NAPLAN numeracy scores and point estimates for the variance components (VC) at levels 3, 2 and 1.

| Model | Regression coefficient (SE) | VC- school level | VC-individual level | Residual error |
| --- | --- | --- | --- | --- |
| Equation (1) | 0.023 (0.038) | - | 0.278 | 0.253 |
| Equation (7) | 0.020 (0.037) | 0.044 | 0.238 | 0.252 |

# S2: Directed acyclic graph (DAG) for the substantive analysis.


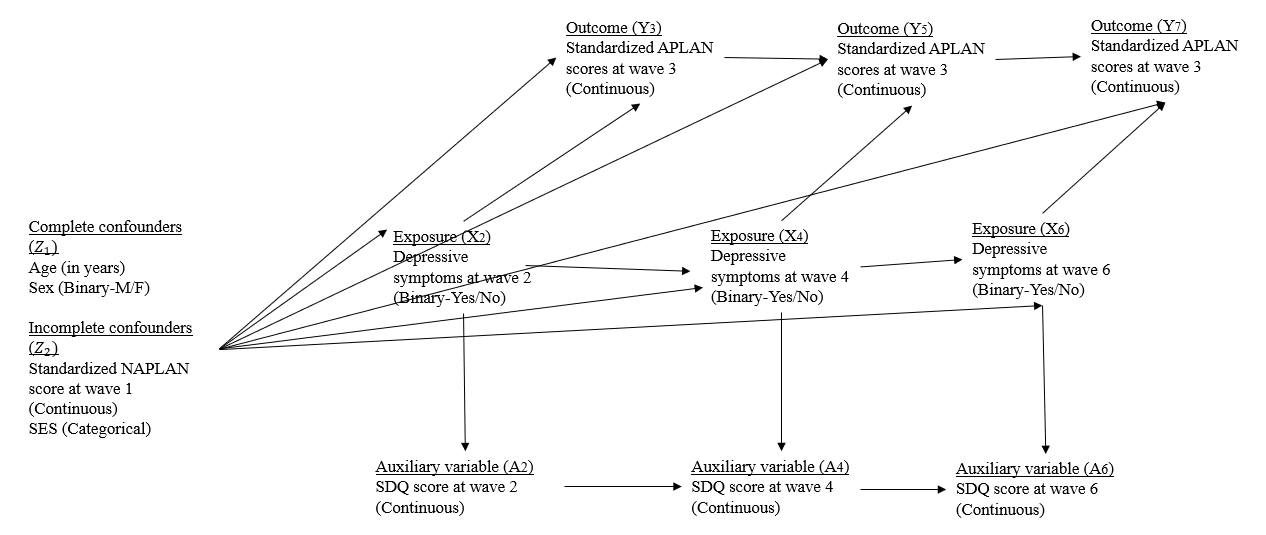


Figure A1: Directed acyclic graph illustrating the causal assumptions in the substantive analysis

# S3: Simulation of the dataset used in this tutorial

## Simulation of complete data

First we generated 40 school clusters which were populated to contain a varying number of students ranging from 8-66 students, similar to the CATS data [1]. The school cluster sizes$\left( {8 \leq n}_{i}\leq66 \right)$ were assumed to follow a truncated log-normal distribution and cluster size for each school $i$ was sampled randomly from this distribution. In order to set the total number of students across the 40 schools to be 1200, the sampled cluster sizes were multiplied by a factor of $1200/\sum_{i=1}^{40} n_{i}$ and rounded to derive a scaled class size. If the total of these scaled class sizes was less than 1200, the deficit was added to the last school cluster, if the total of scaled class size was higher than 1200, the excess was deducted from the last school cluster.

The rest of the variables were then generated sequentially as described below for individual $j$ in cluster$i$. The values of the parameters indexing these distributions were determined by estimating the respective quantity from the CATS data.

1. Child’s age at wave 1 (${age}_{ij1})$was generated from a uniform distribution, $U\left( 7,10 \right).$
2. Child’s sex ${(sex}_{ij})$ was generated by randomly assigning $50\%$of students to be female.
3. Child’s SES quintile at wave 1$({SES}_{ij1}$) was generated by randomly assigning10%,10%,20%, 30% and 30% of respondents to SES quintiles 1,2,3,4 and 5 respectively.
4. The standardised NAPLAN scores at wave 1 ($Y_{ij1}$) were generated from a linear regression model conditional on child’s sex, child’s age at wave 1 and child’s SES quintile:

|  | $Y_{ij1}= -1.2+0.22*\left[ {sex}_{ij}=1 \right]+ 0.08{*age}_{ij1}+$  $0.01*\left[ {SES}_{ij1}=quintile 2 \right]+ 0.37*\left[ {SES}_{ij1}=quintile 3 \right]+$  $0.33*\left[ {SES}_{ij1}=quintile 4 \right]+0.65*\left[ {SES}_{ij1}=quintile 5 \right]+\psi_{ij}$ | (2) |
| --- | --- | --- |

where $\psi_{ij} \begin{matrix} iid \\ \sim\end{matrix} N (0, 1)$

1. Child’s depression status (${depression}_{ijk}$) at waves $k=$2, 4 and 6 was generated using a GLMM conditional on child’s age at wave 1, child’s sex, NAPLAN scores at wave 1, child’s SES quintile and wave:

|  | $logit\left\{ P\left( {depression}_{ijk}=1 \right) \right\}= -4.0+ 0.31*{age}_{ij1}+0.08*k+$  $\left( -0.52 \right)*{[sex}_{ij}=1] +(-0.05)*Y_{ij1}+$  $\left( -0.3 \right)*\left[ {SES}_{ij1}=quintile 2 \right]+\left( -0.4 \right)*\left[ {SES}_{ij1}=quintile 3 \right]+$  $\left( -0.57 \right)*\left[ {SES}_{ij1}=quintile 4 \right]+\left( -0.86 \right)*\left[ {SES}_{ij1}=quintile 5 \right]+ u_{0i}+u_{0ij}$ | (3) |
| --- | --- | --- |

where $u_{0ij}$ and $u_{0i}$ are distributed as $u_{0ij}\begin{matrix} iid \\ \sim\end{matrix}N\left( 0,{1.5}^{2} \right), u_{0i}\begin{matrix} iid \\ \sim\end{matrix} N(0,{0.25}^{2})$ respectively.

1. We then generated the auxiliary variable, child’s behavioural problems at waves 2, 4 and 6 (${SDQ}_{ijk}$), which is not included in the analysis model but is associated with the exposure, using a LMM conditional on depression symptoms at waves 2, 4 and 6 and wave:

|  | ${SDQ}_{ijk}=16+1.6*{depression}_{ijk}++(-0.1)*{wave}_{ijk}+\nu_{0i}+\nu_{0ij}+\epsilon_{ijk}$ | (6) |
| --- | --- | --- |

where$\epsilon_{ijk}$, $\nu_{0i}$ and $\nu_{0ij}$ are iid as; $\epsilon_{ijk}\sim N \left( {0,3.0}^{2} \right)$ $\nu_{0i}\sim N\left( 0,{0.8}^{2} \right),$and $\nu_{0ij}\sim N({0,4.0}^{2}$) respectively.

1. Finally child’s standardized NAPLAN score$(Y_{ijk})$ at waves $k=$ 3, 5 and 7 was generated from a LMM as shown below:

|  | $Y_{ijk}= 2.0 +\left( -0.02 \right)*{depression}_{ij\left( k-1 \right)}+$  $\left( -0.01 \right)*k+\left( -0.2 \right)*{age}_{ij1}+ 0.15*\left[ {sex}_{ij}=1 \right]+$  $0.7*Y_{ij1}+\left( -0.02 \right)*\left[ {SES}_{ij1}=quintile 2 \right]+$  $\left( -0.10 \right)*\left[ {SES}_{ij1}=quintile 3 \right]+\left( 0.02 \right)*\left[ {SES}_{ij1}=quintile 4 \right]+\left( -0.02 \right)*\left[ {SES}_{ij1}=quintile 5 \right]+{\left( -0.01 \right)*SDQ}_{ijk}+a_{0i}+a_{0ij}+\varepsilon_{ijk}$ | (4) |
| --- | --- | --- |

where $\varepsilon_{ijk}\begin{matrix} iid \\ \sim\end{matrix} N \left( {0.25}^{2} \right)$ and $a_{0i}$ and $a_{0ij}$ are distributed as $a_{0i}\begin{matrix} iid \\ \sim\end{matrix}N\left( {0,0.05}^{2} \right), a_{0ij}\begin{matrix} iid \\ \sim\end{matrix}N({0,0.25}^{2}$) respectively.

## Simulation of missing data

To simulate missingness, data were set to missing in depressive symptom scores at waves 2, 4 and 6 (the exposure of interest), NAPLAN scores at wave 3,5 and 7 (the outcome), SDQ values at waves 2,4 and 6, SES and NAPLAN scores at baseline (wave 1), as detailed below.

1. Missing values in SES and NAPLAN scores at baseline were generated by drawing from a logistic regression model dependent on age at baseline and sex as shown below:

|  | $\mathrm{logit}\left\{ P(M_{SES_{ij1}}=1) \right\}=-1.5+0.03*{age}_{ij1}+0.01*{sex}_{ij}$ | (5) |
| --- | --- | --- |

|  | $\mathrm{logit}\left\{ P(M_{Y_{ij1}}=1) \right\}=-2.1+0.05*{age}_{ij1}+0.02*{sex}_{ij}$ | (6) |
| --- | --- | --- |

1. Missing values in depressive symptom scores at waves 2, 4 and 6 were generated by drawing from a GLMM dependent on baseline variables age, wavesex, NAPLAN at wave 1, SES at wave 1, SDQ values (at waves 2,4 and 6), and NAPLAN scores (at waves 3,5 and 7)

|  | $\mathrm{Logit}\left\{ P\left( M_{depression_{ijk}}=1 \right) \right\}=-8.0+ 0.72*{age}_{ij1}+\left( -0.11 \right)*k+ \left( 0.16 \right)*{[sex}_{ij}=1] +(-0.17)*Y_{ij1}+$  $\left( -0.39 \right)*\left[ {SES}_{ij1}=quintile 2 \right]+\left( 0.27 \right)*\left[ {SES}_{ij1}=quintile 3 \right]+$  $\left( 0.19 \right)*\left[ {SES}_{ij1}=quintile 4 \right]+\left( -0.03 \right)*\left[ {SES}_{ij1}=quintile 5 \right]+\left( -0.13 \right)*Y_{\mathrm{ij}\left( k+1 \right)}++0.04*\mathrm{SDQ}_{\mathrm{ijk}}+ u_{0i}+u_{0ij}$ | (7) |
| --- | --- | --- |

where $u_{0ij}$ and $u_{0i}$ are distributed as $u_{0ij}\begin{matrix} iid \\ \sim\end{matrix}N\left( 0,{0.05}^{2} \right), u_{0i}\begin{matrix} iid \\ \sim\end{matrix} N(0,{0.01}^{2})$ respectively.

1. Missing values in NAPLAN scores at waves 3,5 and 7 were generated by drawing from a GLMM dependent on baseline variables age, wave, sex, NAPLAN at wave 1, SES at wave 1, SDQ values (at waves 2,4 and 6), and depression scores (at waves 2,4,6)

|  | $\mathrm{Logit}\left\{ P\left( {M_{NAPLAN}}_{ijk}=1 \right) \right\}=-23+ 1.77*{age}_{ij1}+0.7*k+ 0.01*{[sex}_{ij}=1] +(-0.70)*Y_{ij1}+\left( -4.9 \right)*\left[ {SES}_{ij1}=quintile 2 \right]+\left( -1.9 \right)*\left[ {SES}_{ij1}=quintile 3 \right]+$  $\left( 2.19 \right)*\left[ {SES}_{ij1}=quintile 4 \right]+\left( -2.35 \right)*\left[ {SES}_{ij1}=quintile 5 \right]+\left( -0.25 \right)*\mathrm{depression}_{\mathrm{ij}\left( k-1 \right)}++0.11*\mathrm{SDQ}_{ij(k-1)}+ u_{0i}+u_{0ij}$ | (8) |
| --- | --- | --- |

where $u_{0ij}$ and $u_{0i}$ are distributed as $u_{0ij}\begin{matrix} iid \\ \sim\end{matrix}N\left( 0,{2.0}^{2} \right), u_{0i}\begin{matrix} iid \\ \sim\end{matrix} N(0,{0.4}^{2})$ respectively.

# S4 Sub-functions within jomo() wrapper function in the R package jomo

| **Higher-level wrappers** |  | jomo | | | | |
| --- | --- | --- | --- | --- | --- | --- |
|  | Type of variables | jomo1 | jomo1ran | | | jomo2 |
| **Description** |  | MVNI    Not tailored for clustered data | MVNI  + Random effects  + No missing  level 2 variables  + Fixed cluster-specific level-1 covariance | MVNI  + Random effects  + No missing  level 2 variables  + Fixed/Random  cluster-specific level-1 covariance | MVNI  + Random effects  + Missing  level 2 variables  + Common/Fixed/ Random cluster-specific level-1 covariance | |
| **Sub-functions** | Binary/categorical | jomo1cat | jomo1rancat | jomo1rancathr | jomo2com | |
|  | Continuous | jomo1con | jomo1rancon | jomo1ranconhr | jomo2hr | |
|  | Mixed | jomo1mix | jomo1ranmix | jomo1ranmixhr |  | |

Table 1: Summary of sub functions used by the jomo() wrapper function, given the type of model specification (e.g., not clustered or clustered) and the variable type being imputed

# S5: Results and computational time for MI methods

## MI approaches for longitudinal data

Point estimate (and standard error) for the effect of early depressive symptoms on subsequent standardized NAPLAN numeracy scores and the estimates for the variance components (VC).

***Using R***

| Method | Regression coefficient (SE) | VC- individual level | Residual error | Computational time (minutes) |
| --- | --- | --- | --- | --- |
| JM-1L-wide | -0.005 (0.034) | 0.284 | 0.261 | 36.96 |
| FCS-1L-wide | -0.008 (0.033) | 0.280 | 0.262 | 1.52 |
| FCS-1Lwide-MTW | -0.012 (0.034) | 0.327 | 0.285 | 1.37 |
| JM-2L | -0.039 (0.052) | 1.199 | 0.585 | 14.35 |
| FCS-2L | -0.010 (0.034) | 0.295 | 0.264 | 14.92 |

***Using Stata***

| Method | Regression coefficient (SE) | VC- individual level | Residual error | Computational time (minutes) |
| --- | --- | --- | --- | --- |
| JM-1L-wide | -0.002 (0.032) | 0.283 | 0.260 | 1.62 |
| FCS-1L-wide | -0.007 (0.034) | 0.280 | 0.261 | 3.36 |
| FCS-1Lwide-MTW | -0.013 (0.036) | 0.273 | 0.275 | 3.79 |

## MI approaches for longitudinal data with higher-level clustering

Point estimate (and standard error) for the effect of early depressive symptoms on subsequent standardized NAPLAN numeracy scores and the estimates for the variance components (VC).

***Using R***

| Method | Regression coefficient (SE) | VC- school level | VC-individual level | Residual error | Computational time (minutes) |
| --- | --- | --- | --- | --- | --- |
| JM-1L-DI-wide* | -0.017 (0.034) | 0.056 | 0.236 | 0.267 | 63.39 |
| FCS-1L-DI-wide* | -0.012(0.034) | 0.055 | 0.239 | 0.266 | 4.37 |
| JM-2L-wide | -0.008 (0.037) | 0.048 | 0.239 | 0.272 | 55.91 |
| FCS-2L-wide* | -0.007 (0.038) | 0.048 | 0.239 | 0.264 | 636.0 |
| JM-2L-DI* | -0.146 (0.041) | 0.057 | 0.435 | 0.359 | 290.67 |
| FCS-2L-DI** | - | - | - | - | - |
| FCS-3L^+^ | -0.015(0.034) | 0.046 | 0.241 | 0.260 | 3.29 |

*** The imputation model converged, post imputation analysis in one of the imputed datasets generated non-convergence warnings.

** The imputation model didn’t converge (terminated with an error)

+ Singular fit warnings were generated for 13 (out of 660) imputation iterations, post imputation analysis in one of the imputed datasets did not achieve convergence.

***Using Stata***

| Method | Regression coefficient (SE) | VC- school level | VC-individual level | Residual error | Computational time (minutes) |
| --- | --- | --- | --- | --- | --- |
| JM-1L-DI-wide | -0.007(0.031) | 0.053 | 0.236 | 0.266 | 3.65 |
| FCS-1L-DI-wide | -0.013 (0.035) | 0.055 | 0.236 | 0.267 | 5.55 |

1. Mundy LK, Simmons JG, Allen NB, Viner RM, Bayer JK, Olds T, Williams J, Olsson C, Romaniuk H, Mensah F: **Study protocol: the childhood to adolescence transition study (CATS)**. *BMC pediatrics* 2013, **13**(1):160.

2. Quartagno M, Carpenter JRJBj: **Multiple imputation for discrete data: Evaluation of the joint latent normal model**. 2019, **61**(4):1003-1019.

3. Carpenter J, Kenward M: **Multiple imputation and its application**: John Wiley & Sons; 2012.

4. Enders CK, Keller BT, Levy R: **A fully conditional specification approach to multilevel imputation of categorical and continuous variables**. *Psychological methods* 2018.
